# 

**Published:** 1895-09

**Authors:** 


### BOOKS RECEIVED.

Remote Consequences of Injuries of the Nerves and Their Treatment. An examination of the present condition of wounds received in 1863-65, with additional illustrative cases. By John K. Mitchell, M. D., Assistant Physician to the Orthopedic Hospital and Infirmary for Nervous Diseases, Philadelphia; Lecturer on Physical Diagnosis in the University of Pennsylvania. In one handsome 12mo volume of 233 pages, with twelve illustrations. Cloth, $1.75. Philadelphia: Lea Brothers & Co. 1895.

The Johns Hopkins Hospital Reports. Report in Gynecology, III., Volume IV., Number 7-8. Baltimore: The Johns Hopkins Press. 1895.

A System of Surgery. By American Authors. Edited by Frederic S. Dennis, M, D., Professor of the Principles and Practice of Surgery,

Bellevue Hospital Medical College, New York; President of the American Surgical Association, etc., assisted by John S. Billings, M. D., LL. D., D. C. L., Deputy Surgeon-General, U. S. A. To be completed in four imperial octavo volumes, containing about 900 pages, each with index. Volume II., 915 pages, 515 engravings and ten colored plates. Price, per volume, $6.00 in cloth; $7.00 in leather; $8.50 in half morocco, gilt back and top. Philadelphia: Lea Brothers & Co. 1895.

The History of Prostitution: Its Extent, Causes and Effects throughout the World. By William W. Sanger, M. D., Resident Physician, Blackwell's Island, N. Y. Octavo, pp. 709. New York: The American Medical Press, 816 Broadway. 1895.

Skiascopy and its Practical Application to the Study of Refraction. By Edward Jackson, A. M., M. D., Professor of Diseases of the Eye in the Philadelphia Polyclinic; Surgeon to Wills' Eye Hospital, etc., etc. Pp. 112, with twenty-six illustrations. Price, $1.00. Philadelphia: The Edwards & Docker Co., 518 Minor street. 1895.

A Treatise on the Nervous Diseases of Children, for Physicians and Students. By B. Sachs, M. D., Professor of Mental and Nervous Diseases in the New York Polyclinic; Consulting Neurologist to the Mt. Sinai Hospital; Neurologist to the Montefiore Home for Chronic Invalids; ex-President of the American Neurological Association. One volume, pp. 688, 8vo, illustrated by 169 engravings, in black and color and a colored plate. Muslin, $5.00. New York: William Wood & Company. 1895.

Modern Medicine and Homeopathy. By John B. Roberts, A. M., M. D., ex-President of the Philadelphia County Medical Society and of the Medical Society of the State of Pennsylvania. Cloth, 16mo, pp. 72. Price, 75 cents. Philadelphia: The Edwards & Docker Company, 518-520 Minor street. 1895.

The American Academy of Railway Surgeons. Official report of first meeting held at Chicago, Ill., November 9-10, 1895. Edited by R. Harvey Reed, M. D., Columbus, O. Chicago: American Medical Association Press. 1895.

The Pocket Materia Medica and Therapeutics. A Résumé of the Action and Doses of all Officinal and Non-officinal drugs now in common use. By C. Henri Leonard, A. M., M. D., Professor of the Medical and Surgical Diseases of Women and Clinical Gynecology in the Detroit College of Medicine; Member of the American Medical Association, etc., etc. Second edition, revised and enlarged. Cloth, large 16mo, 367 pages. Price, post-paid, $1.00. Detroit: The Illustrated Medical Journal Co., Publishers. 1895.

Exercise and Food for Pulmonary Invalids. By Charles Denison, A. M., M. D., Denver, Col., Professor of Diseases of the Chest and of Climatology, University of Denver, etc. Price, 35 cents. Denver: The Chain & Hardy Company. 1895.

Formulaire des spécialités pharmaceutiques, composition, indications thérapeutiques. mode d'emploi et dosage, à l'usage des médecins, par le D M. Gautier, ancien interne des hôpitaux, et F. Renault, pharmacien de 1re classe, lauréat de l'École de pharmacie. 1 vol. in-18 de 300 pages, cartonné 3 fr. Librairie J.-B. Baillière et Fils, 19 rue Hautefeuille (près du boulevard Saint-Germain), à Paris. 1895.

Seventeenth Annual Report of the State Board of Health of Illinois. Being for the year ended December 31, 1894. With an appendix containing the official register of physicians and midwives, 1895. Springfield, Ill.; Ed. F. Hartman, State Printer. 1895.

Practical Dietetics with Special Reference to Diet in Disease. By W. Gilman Thompson, M. D., Professor of Materia Medica, Therapeutics and Clinical Medicine in the University of the City of New York; Visiting Physician to the Presbyterian and Bellevue Hospitals, New York. Large 8vo, 800 pages. Illustrated. Prices, cloth, $5.00; sheep, $6.00. Sold by subscription only. New York: D. Appleton & Co., Publishers, 72 Fifth avenue. 1895.

Annual Report of the Department of Health of the City of Chicago for the year ending December 31, 1894. Arthur R. Reynolds, M. D., Commissioner of Health, Chicago. 1895.

The Science and Art of Obstetrics. By Theophilus Parvin, M. D., LL. D., Professor of Obstetrics and the Diseases of Women and Children in Jefferson Medical College, Philadelphia. New (third) edition. In one very handsome 8vo volume of 677 pages, with 267 engravings and two colored plates. Cloth, $4.25; leather, $5.25, Philadelphia: Lea Brothers & Co., Publishers. 1895.

